# Extracellular vesicles and Alzheimer’s disease: hotspots, trends, and future directions—a tri-database bibliometric analysis

**DOI:** 10.3389/fnagi.2026.1951959

**Published:** 2026-10-26

**Authors:** Xi Wang, Bing-Wei Zhang

**Affiliations:** 1 Department of Neurology, The First Affiliated Hospital of Dalian University, Dalian, China; 2 Department of Neurology, Anshan Central Hospital, Anshan, Liaoning, China

**Keywords:** Alzheimer’s disease, bibliometrics, CiteSpace, extracellular vesicles, multi-database analysis, VOSviewer

## Abstract

**Background:**

Alzheimer’s disease is a neurodegenerative disorder marked by progressive cognitive decline. The pathogenesis of AD is complex, and effective methods for early diagnosis and treatment are currently lacking. Extracellular vesicles, which are key mediators of intercellular communication, significantly influence the onset and progression of AD. Consequently, a systematic review of this field is essential.

**Methods:**

This study utilized the WoSCC and Scopus databases to gather English “Articles” and “Reviews.” Following duplicate removal via Python, bibliometric methods systematically analyzed research hotspots and frontiers concerning extracellular vesicles in Alzheimer’s disease. The analysis encompassed publication trends, countries, institutions, authors, journals, keywords, and references. Concurrently, the PubMed database was searched to verify the robustness and consistency of the analysis results.

**Results:**

This study conducted a bibliometric analysis of 2,626 articles, with 1,614 articles from PubMed used for consistency verification. The findings indicate a continuous rise in research interest in this field. Key areas of focus include emerging pathological mechanisms, peripheral blood biomarkers, targeted drug delivery, miRNA, and clinical trials. Cross-database verification confirmed a strong consistency in publication trends, countries, institutions, and authors, with a significant overlap of keywords and prominent research hot spots.

**Conclusion:**

This study demonstrates a shift in the research focus on Extracellular vesicles within Alzheimer’s disease from single-pathology investigations to exploring multiple mechanisms. Their significance in precise diagnosis and targeted therapy is increasingly evident. However, clinical trials remain limited. Future efforts should prioritize enhancing clinical validation and standardizing isolation techniques.

## 1 Introduction

Alzheimer’s disease (AD) is a neurodegenerative disease characterized by progressive memory impairment and cognitive decline (Gustavsson et al., 2023). With the accelerating global aging process, the prevalence and incidence of AD are increasing year by year, making it a significant disease that seriously threatens public health and socioeconomic development (Alzheimer’s Dementia, 2023). The core pathological features of AD are widely recognized as Amyloid-beta (Aβ) plaque aggregation and neurofibrillary tangle formation. In addition, multiple mechanisms such as insulin resistance, oxidative stress, and neuroinflammation have also been proven to be involved in the occurrence and development of the disease (De Strooper and Karran, 2016). Despite the numerous advancements in research on the pathogenesis of AD, there is still a lack of effective early diagnosis, precise disease staging criteria, and disease-modifying treatment strategies that can halt the disease progression in clinical practice.

Extracellular vesicles (EVs) are cell-derived phospholipid-bilayer vesicles, mainly including exosomes, microvesicles, and apoptotic bodies (Hessvik and Llorente, 2018). EVs are widely present in various body fluids such as blood, cerebrospinal fluid, urine, and saliva. They can carry bioactive molecules such as proteins, lipids, and nucleic acids, and play important roles in intercellular information transmission and disease regulation (Canseco-Rodriguez et al., 2022). A large number of studies have shown that EVs have dual roles in the pathological processes related to Alzheimer’s disease. On the one hand, EVs have neuroprotective and immunomodulatory effects. They can act as natural carriers to transport Aβ and Tau proteins to glial cells for degradation, thereby reducing the pathological burden (Garcia-Contreras and Thakor, 2023). On the other hand, EVs can also mediate the spread of pathological proteins between neurons, thus accelerating the progression of the disease (Asai et al., 2015; Li et al., 2022). In addition, due to their unique lipid bilayer structure, EVs can penetrate the blood—brain barrier and have the ability to carry “cargo,” which makes neuron—derived EVs from peripheral blood show important application potential in the early diagnosis and disease detection of AD (Fiandaca et al., 2015). Meanwhile, biologically active molecules with therapeutic effects carried by EVs can also penetrate the blood—brain barrier and reach the intracranial lesions through peripheral circulation injection, suggesting that EVs have broad application prospects in the treatment and prevention of AD (Zhao et al., 2021).

With the continuous deepening of research, studies on the mechanism of action, diagnostic value, and therapeutic value of EVs in AD have grown rapidly. Currently, research in this field covers a wide range of topics and directions. A systematic summary and quantitative analysis can quickly provide an understanding of the evolutionary process and development trends of research in this field. Bibliometrics is a method based on mathematics and statistics. By analyzing the number of documents, authors, institutions, keywords, and citation relationships, it can provide a detailed and systematic overview of the knowledge structure and emerging trend (Diem and Wolter, 2013). Xiaolian Xing team conducted a bibliometric analysis of 602 documents in this field published from 2001 to 2023 (Xing et al., 2024). However, new research hotspots have emerged in the past 2 years, and further updated and more comprehensive bibliometric analysis is still needed to reveal the latest research hotspots and development trends. Based on this, this study used the Web of Science Core Collection (WOSCC) and Scopus databases as the data sources for bibliometric analysis and used the PubMed database for consistency verification. Meanwhile, bibliometric tools such as CiteSpace, VOSviewer, and Origin were used to conduct a systematic analysis of relevant research in this field, aiming to reveal research hotspots and development trends and provide references for subsequent basic research and clinical translation.

## 2 Materials and methods

### 2.1 Data sources and collection

This study used the WOSCC, Scopus, and Pubmed databases as the sources of literature data. WOSCC is one of the most widely used databases globally, featuring high—quality citation data and a well—structured citation system (Liao et al., 2026). Scopus is one of the largest peer—reviewed literature databases globally, with extensive multidisciplinary coverage and systematically organized bibliographic information (Martín-Martín et al., 2021). The Pubmed database is an authoritative database in the field of medical biology, which includes numerous high—impact—factor literatures (Tang et al., 2026). The retrieval strategies are as follows: (1) The publication time was limited up to April 30, 2026; (2) The language was limited to English; (3) The document types were limited to Article and Review; (4) The same search terms were used and adjusted according to the grammar requirements of each database. The specific retrieval strategies are detailed in Supplementary Table 1. After the initial retrieval, Python (version 3.11) was used to clean and integrate the data of literatures from the Woscc and Scopus databases: For literatures with DOIs, duplicates were removed based on DOIs; for literatures without DOIs, duplicates were removed based on titles. Finally, Articles and Reviews were selected. The literature screening process and the bibliometric analysis process will be illustrated by figures later. It should be noted that since the data collection deadline was April 30, 2026, articles published in 2026 have not been fully included. Therefore, the number of articles published in 2026 may be underestimated. The literature screening process and the bibliometric analysis process are shown in Figure 1.

**Figure 1:**
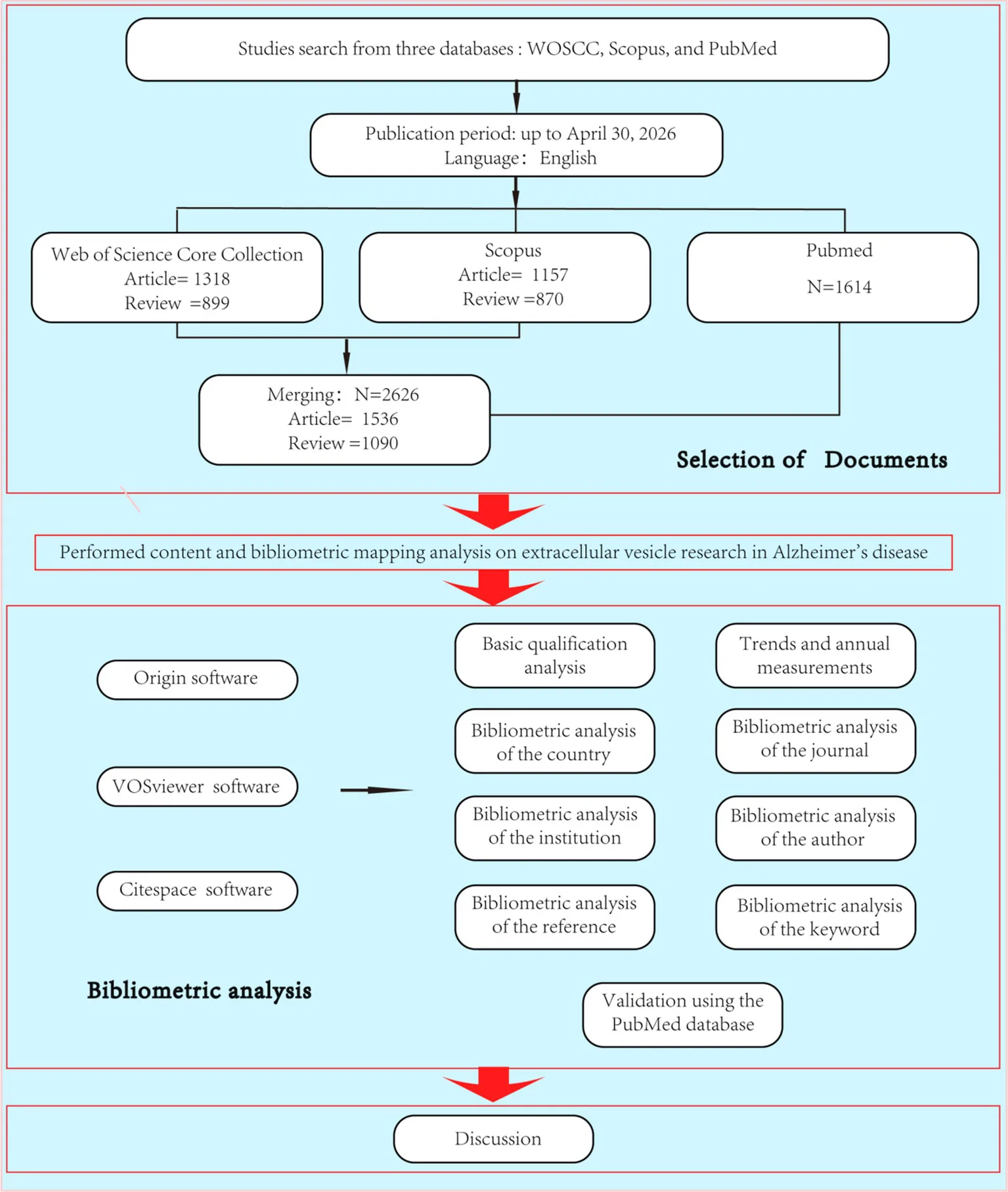
Flowchart of literature screening and bibliometric analysis.

### 2.2 Data analysis

In this study, three bibliometric tools were employed to conduct a systematic analysis of the included literature. First, Origin 2026 (Version 10.3) was used to analyze the annual publication trends, journals, institutions, and authors. Second, VOSviewer (Version 1.6.20) was used to perform a visual analysis of countries, journals, institutions, authors, and keywords to reveal the cooperation network structure and distribution characteristics among different research subjects (Hu et al., 2026). Finally, CiteSpace (Version 6.4.R1), developed by Professor Chaomei Chen, was used to analyze keywords and references, with a focus on identifying the evolutionary characteristics of research hotspots and frontiers (Zhang G. et al., 2026). The above-mentioned tools enabled a multi-dimensional analysis, identifying key trends, hotspots, and future directions for EVs in AD research.

### 2.3 Validation in the PubMed database

To evaluate the robustness and comprehensiveness of the analysis results based on the WOSCC and Scopus databases, the PubMed database was used in this study for consistency verification. The retrieval strategy was completely equivalent to that for the WOS and Scopus databases, ensuring that the keywords, time range, and language restrictions remained consistent. By systematically comparing the similarities and differences in aspects such as literature publication trends, major research countries, core institutions, high—yielding authors, and the distribution of high—frequency keywords, the reliability of the core conclusions of this study was tested.

## 3 Results

### 3.1 Basic qualification analysis

Through retrieval, 1,318 Articles and 899 Reviews were initially obtained from WOSCC, and 1,157 Articles and 870 Reviews were initially obtained from Scopus. After removing duplicates, 1,536 Articles and 1,090 Reviews were selected, and a total of 2,626 articles were finally included, with 2,222 from WOSCC and 404 from SCOPUS. These publications came from 3,475 institutions across 78 countries, involved 13,848 authors, appeared in 752 journals, and cited 150,252 references.

### 3.2 Trends and annual measurements

As shown in Figure 2A, regarding the research on EVs in AD, the number of published papers was relatively small from 1991 to 2011, with no more than 20 papers published each year. Since 2012, the number of publications has begun to gradually increase. Especially from 2020 to 2026, the annual number of publications exceeded 200, indicating that the research on EVs in AD has received increasing attention. It is worth noting that the number of publications in the first 4 months of 2026 has exceeded one-third of the total number of publications in 2025, suggesting that the research enthusiasm in this field continues to rise.

**Figure 2:**
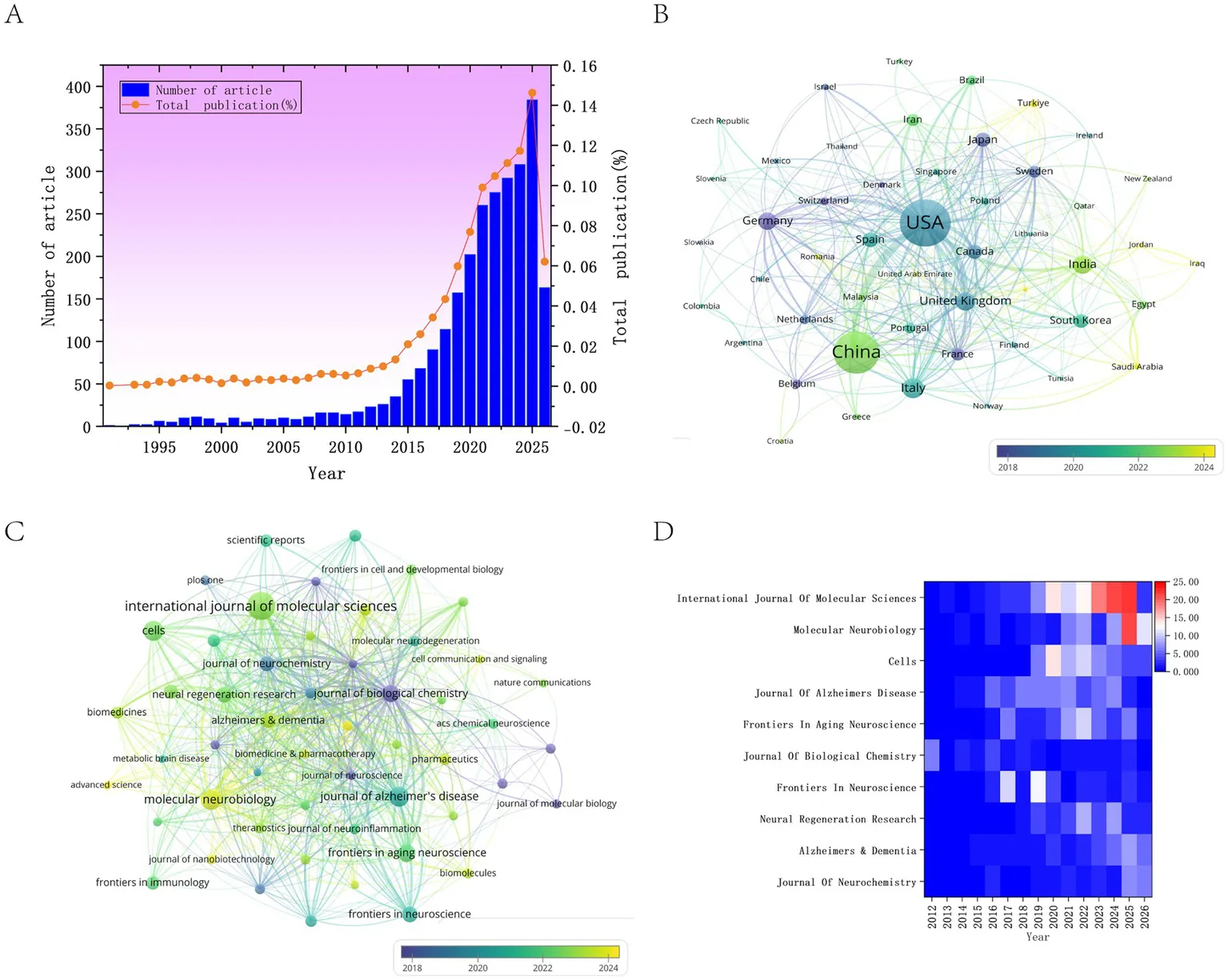
**(A)** Dot-line-bar chart of the annual publication volume from 1991 to 2026. **(B)** Visual map of the country overlay time network spanning from 1991 to 2026. **(C)** Visual map of the journal overlay time network spanning from 1991 to 2026. **(D)** Heatmap of journals from 2012 to 2026.

### 3.3 Bibliometric analysis of the country

Based on the bibliometric data, 78 countries have been involved in this research area. Table 1 presents the top 10 countries by publication volume. The United States is the country with the highest number of publications (904) and the highest number of citations (63928), demonstrating strong academic influence. Although the number of publications in the United Kingdom and Germany is relatively small, the average number of citations is high, indicating that although these countries have a lower number of publications, each article has a relatively high academic influence. In the visualization analysis of the national overlay time network, the size of the circular node represents the number of publications; the connection between nodes represents the strength of association, and the color represents the average active years. The nodes of countries such as Germany, Japan, and France are purple, indicating that these countries started research in this field earlier and the average publication year of their core literature is earlier. The node of China is yellow**—**green, indicating that the research results are mainly concentrated in recent years. Meanwhile, China has the second**—**highest number of publications (D = 741), indicating that China has become an emerging force in this field (Figure 2B).

**Table 1:** The top 10 countries in terms of the number of publications.

Rank	Country	Documents	Citations	Avg. citations	Total link strength
1	USA	904	63,928	70.7168	339
2	China	741	26,774	36.1323	132
3	Italy	183	8,312	45.4208	81
4	India	155	2,749	17.7355	66
5	United Kingdom	153	15,581	101.8366	110
6	Germany	140	13,393	95.6643	82
7	Spain	100	4,603	46.03	49
8	Japan	96	5,192	54.0833	32
9	Canada	92	5,436	59.087	59
10	South Korea	87	3,364	38.6667	31

### 3.4 Bibliometric analysis of the journal

Among the 752 journals included in the analysis, a total of 122 journals published more than 5 papers. Table 2 lists the top 10 journals in terms of the number of papers published in this field, among which the impact factor of Alzheimers & Dementia exceeds 10. The top three journals ranked by the number of papers published are: International Journal Of Molecular Sciences, Molecular Neurobiology, and Cells. Although journals such as Journal of Biological Chemistry, Alzheimers & Dementia, and Journal of Alzheimers Disease have a slightly lower number of papers published, they have a relatively high link strength, indicating that the articles published in these journals are cited by multiple different fields and journals, and have important academic influence (Figure 2C). In addition, the nodes of International Journal of Molecular Sciences, Molecular Neurobiology, and Cells are yellow to yellow**—**green. Combined with their high number of papers published recently, it indicates that molecular biology remains a research hotspot in this field. The journal heat map (Figure 2D) further shows that the main source journals of EVs in the AD field cover four major disciplinary categories: neuropathology, EVs biology, molecular biology, and nanomedicine, reflecting the distinct interdisciplinary characteristics of this field and the multidisciplinary integration pattern from basic mechanisms to clinical translation.

**Table 2:** The top 10 journals in terms of the number of publications.

Rank	Journal	Documents	Citations	Total link strength	IF (2025)	Country
1	International Journal Of Molecular Sciences	119	4,267	685	6.208	Switzerland
2	Molecular Neurobiology	63	1998	629	5.1	USA
3	Cells	59	3,057	279	6.0	Switzerland
4	Journal of Alzheimers Disease	59	2,135	758	4.0	Netherlands
5	Frontiers In Aging Neuroscience	48	1,541	353	4.8	Switzerland
6	Journal of Biological Chemistry	44	8,546	1,174	5.486	USA
7	Frontiers In Neuroscience	38	2,963	238	4.3	Switzerland
8	Neural Regeneration Research	37	1,117	389	6.1	China
9	Alzheimers & Dementia	33	2,409	848	14	USA
10	Journal Of Neurochemistry	33	1,563	397	4.7	United Kingdom

### 3.5 Bibliometric analysis of the institution

Statistics of all research institutions show that a total of 3,475 institutions participated in the research in this field, among which 76 institutions published 10 or more papers. Table 3 lists the top 10 institutions in terms of the number of publications, including 6 institutions from the United States and 4 from China. The National Institute on Aging ranks first with 56 publications and is also the institution with the highest total citations (Figure 3A). It is followed by the University of California, San Francisco. These institutions together form the core group of high**—**productivity and high**—**impact institutions in this research area (Figure 3B). Although Boston University has a relatively small number of publications, its average citation per paper is as high as 124.0294 times, indicating that this institution has prominent academic influence and high**—**quality research output.

**Table 3:** Top 10 institutions in terms of the number of publications.

Rank	Institution	Documents	Citations	Total link strength	Avg. citations	Country
1	National Institute on Aging	56	6,424	2,592	114.7143	USA
2	University of California, San Francisco	43	4,370	1844	101.6279	USA
3	Harvard Medical School	39	2,600	531	66.6667	USA
4	Capital Medical University	38	1,337	524	35.1842	China
5	Shanghai Jiao Tong University	36	2,913	318	80.9167	China
6	Boston University	34	4,217	906	124.0294	USA
7	Johns Hopkins University	32	1,532	612	47.875	USA
8	University of California, San Diego	30	1960	697	65.3333	USA
9	Zhejiang University	28	1,505	317	53.75	China
10	Fudan University	27	1,202	160	44.5185	China

**Figure 3:**
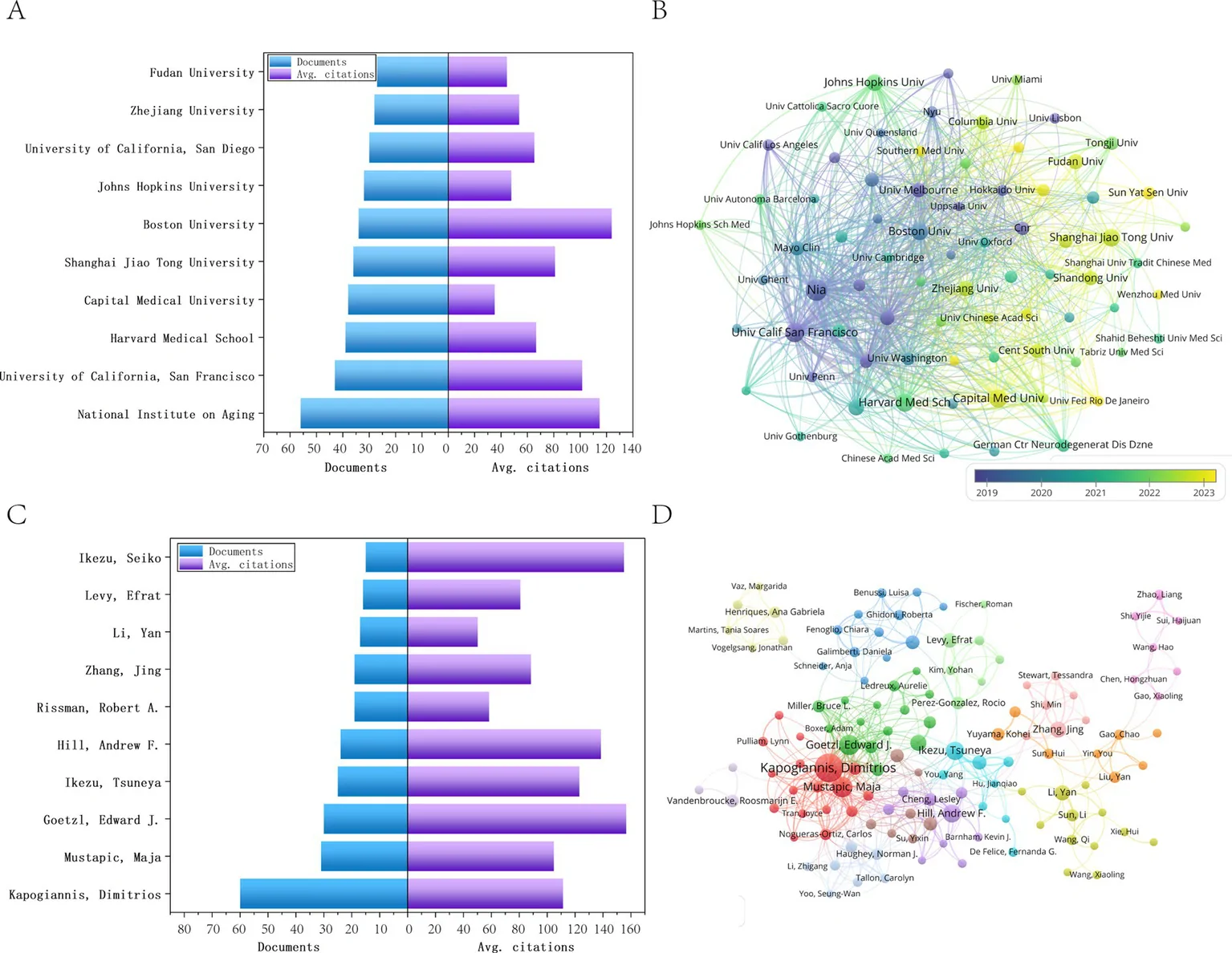
**(A)** Pyramid chart of the top 10 institutions. **(B)** Time-overlay network visualization map of institutions. **(C)** Pyramid chart of the top 10 authors. **(D)** Network visualization map of authors.

### 3.6 Bibliometric analysis of the author

Of the 13,848 authors involved in this field, 211 have published more than 5 articles each. The top 10 most productive authors are listed in Table 4. The top three authors in terms of the number of publications are Dimitrios Kapogiannis, Maja Mustapic, and Edward J Goetzl. Although Andrew F Hill and Seiko Ikezu published relatively fewer articles, they had the highest citation count per article, indicating a high level of influence (Figure 3C). The analysis of the author co**—**occurrence network further showed (Figure 3D) that multiple research clusters centered around scholars such as Dimitrios Kapogiannis, Maja Mustapic, Edward J Goetzl, and Tsuneya Ikezu were formed in this field, presenting the characteristic of “concentration of highly productive core authors and co**—**existence of multiple team collaborations”.

**Table 4:** The top 10 authors in terms of publications.

Rank	Author	Documents	Citations	Total link strength	Avg. citations
1	Dimitrios Kapogiannis	60	6,679	205	111.3167
2	Maja Mustapic	31	3,247	120	104.7419
3	Edward J Goetzl	30	4,698	127	156.6
4	Tsuneya Ikezu	25	3,076	57	123.04
5	Andrew F Hill	24	3,323	56	138.4583
6	Robert A Rissman	19	1,110	20	58.4211
7	Jing Zhang	19	1,679	30	88.3684
8	Yan Li	17	854	25	50.2353
9	Efrat Levy	16	1,293	36	80.8125
10	Seiko Ikezu	15	2,325	37	155

### 3.7 Scientometric analysis of the reference

#### 3.7.1 Citation analysis

Highly cited papers indicate the research directions in the field and have high academic influence. In this study, a total of 134 papers were cited more than 200 times. Supplementary Table 2 lists the top 10 papers in terms of citation times. These papers span from 1998 to 2023 and were all published in international authoritative journals, including 8 research papers (Article) and 2 reviews (Review). According to the research content, these 10 highly cited papers can be summarized into the following three main research directions: (1) The mechanism of action of EVs in AD, including the binding and release of Aβ with EVs (Rajendran et al., 2006), the pathological spread of hyperphosphorylated tau (Asai et al., 2015; Saman et al., 2012), the mechanism of neuroinflammation and microglia (Gao et al., 2023), and axonal lesions and axoplasmic transport disorders (Stokin et al., 2005); (2) EVs as drug delivery systems, such as confirming that targeted EVs can deliver siRNA to the mouse brain (Alvarez-Erviti et al., 2011), and clarifying the effects of cell source, administration route, and targeted modification on the *in vivo* biodistribution of EVs (Wiklander et al., 2015); (3) EVs as a source of biomarkers: systematically summarizing the potential of EVs in the diagnostic field, including the recognition value of exosomal nucleic acid and protein biomarkers in body fluids such as blood and cerebrospinal fluid (Vlassov et al., 2012).

#### 3.7.2 Co-citation analysis

Co-citation reflects the situation where two or more papers are cited simultaneously by subsequent academic works, revealing their intrinsic relevance in research topics (Chen et al., 2022). In this study, 67 co-cited papers have been cited over 100 times. Supplementary Table 3 lists the top 10 papers in terms of co-citation frequency.

Figures 4A,B respectively present the burst analysis results of co-cited documents from 1991 to 2026 and from 2021 to 2026. Figure 4C shows the clustering of co-cited documents from 2021 to 2026 using CiteSpace, and a total of 20 valid clusters were obtained. The clustering results (Q = 0.8612, Silhouette S = 0.9543) have high reliability. According to the research topics, these clusters can be summarized into five main directions: (1) Biomarkers and diagnostic strategies (#2, #3, #8, #11, #16) cover the exploration of brain-derived or peripheral blood-derived EVs and their carried biomarkers in the precise diagnosis, staging, and differential diagnosis of AD; (2) Clinical research and administration routes (#4, #5, #7) involve intranasal administration, intravenous injection, and clinical trials; (3) Treatment-related research (#0, #6, #10, #14, #15) focuses on the sources of therapeutic EVs and the engineering strategy of loading small RNAs; (4) Mechanism-related research (#9, #17, #18) mainly focuses on the emerging pathological mechanisms of AD and their overlap with other neurodegenerative diseases; (5) Methodology and technological progress (#12, #13), focusing on the new separation methods of EVs and the current progress in the field. Overall, the co-citation network clearly reflects the complete research chain from basic mechanism exploration to diagnostic application and then to therapeutic translation.

**Figure 4:**
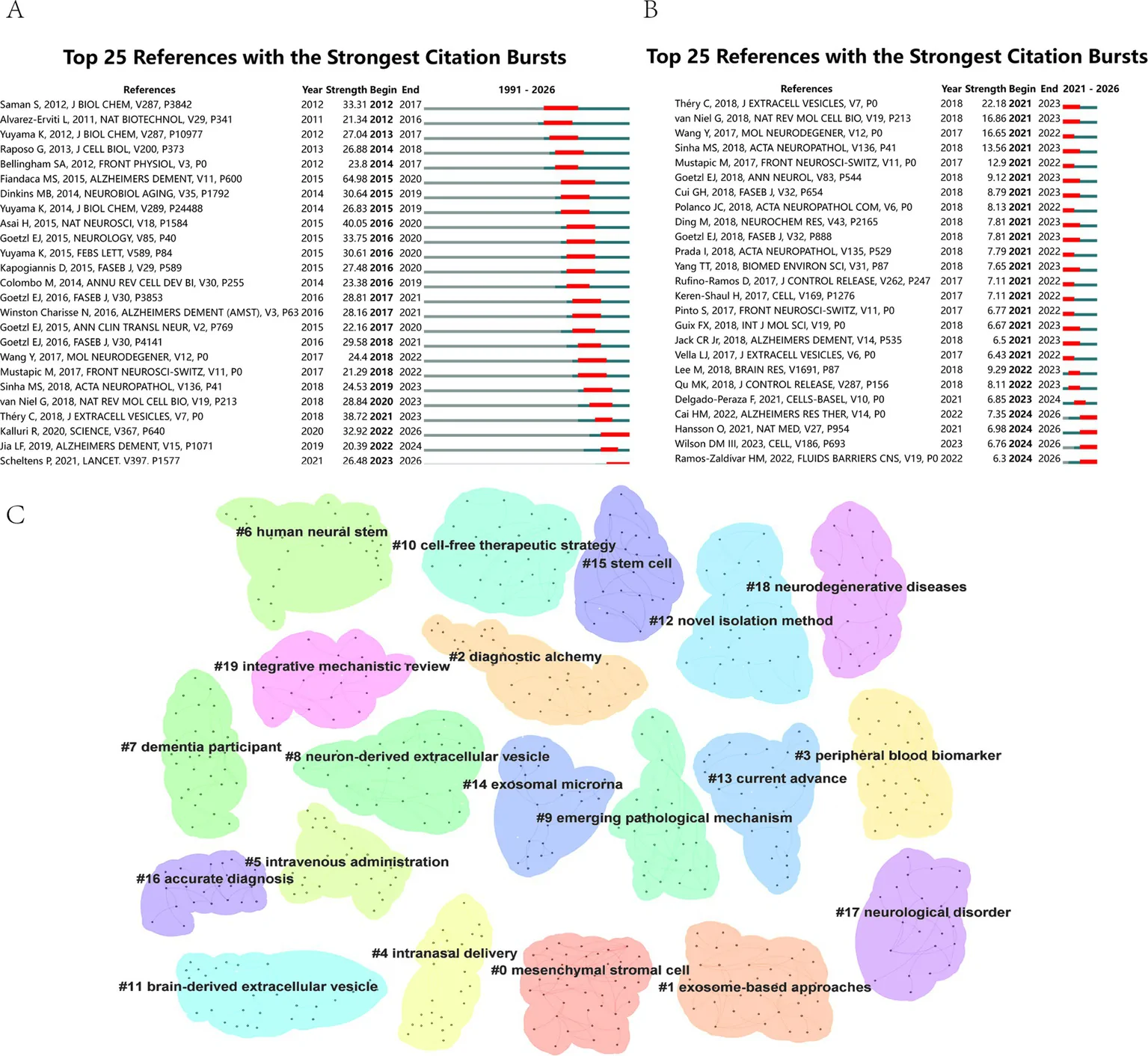
**(A)** Burst detection map of co-cited literature from 1991 to 2026. **(B)** Burst detection map of co-cited literature from 2021 to 2026. **(C)** Clustering of co-cited literature from 2021 to 2026.

### 3.8 Bibliometric analysis of the keyword

Keywords reveal the research theme and content and their frequency of occurrence can be used to measure research hotspots. From 1991 to 2026 the high-frequency keywords in this field mainly include “extracellular vesicles” “Alzheimer’s disease” “tau protein” “β-amyloid protein” etc. (Figures 5A,B). Overall the early research in this field mainly focused on exploring the mechanisms of EVs in the occurrence and development of AD while in the later stage there was an obvious trend of gradual transformation towards clinical treatment.

**Figure 5:**
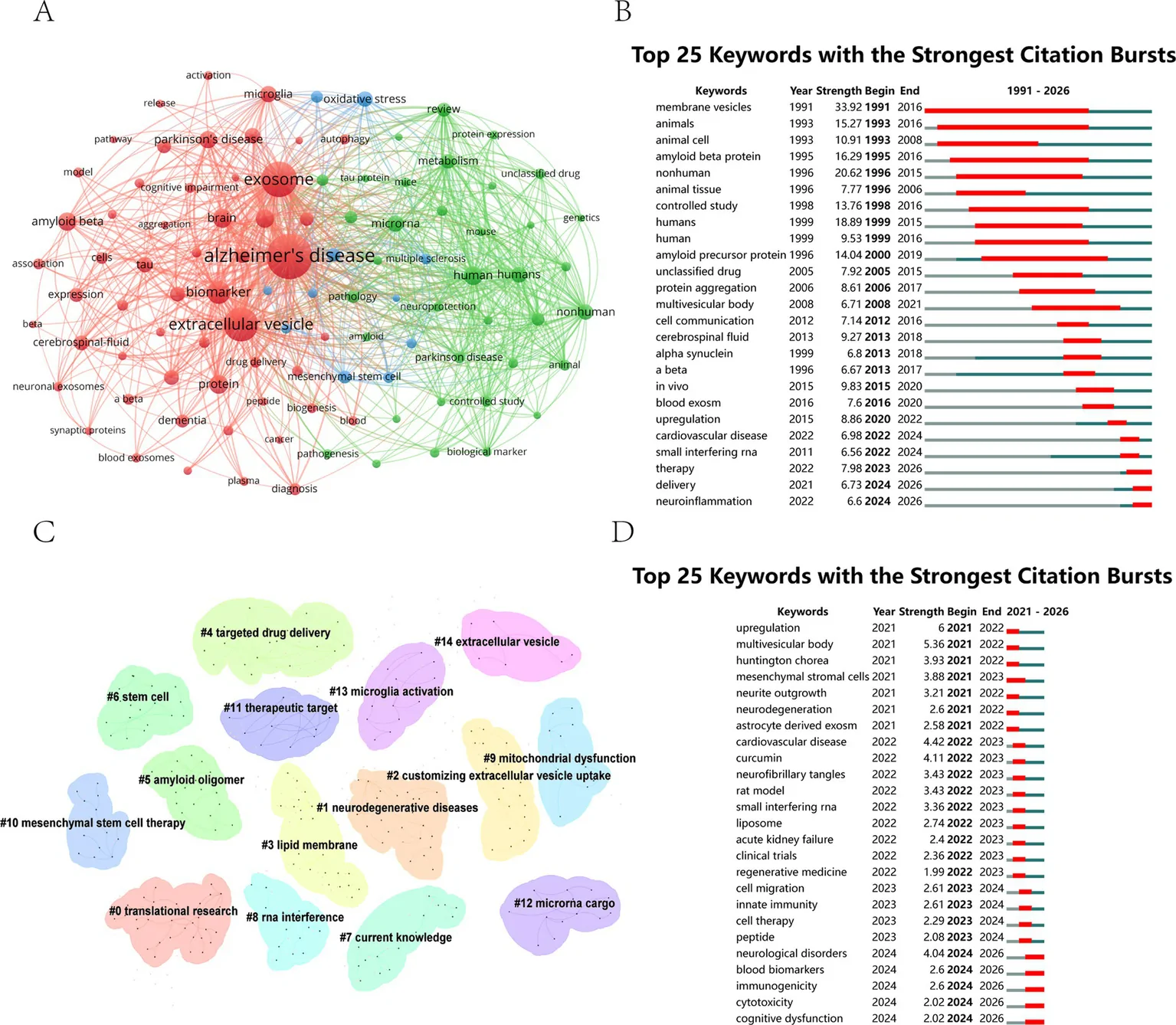
**(A)** Visualization map of keyword networks from 1991 to 2026. **(B)** Keyword burst detection map from 1991 to 2026. **(C)** Keyword clustering map from 2021 to 2026. **(D)** Keyword burst detection map from 2021 to 2026.

The keyword analysis over the past 5 years reflects the dynamic development trends in the field. In this study, CiteSpace was applied to conduct a cluster analysis of the keywords in this field from 2021 to 2026 (Figure 5C). A total of 15 effective clusters were obtained, and the clustering results (Q = 0.827, Silhouette S = 0.9582) had high reliability. According to the research content, the clusters can be summarized into the following four main research directions: (1) Research on basic pathological mechanisms (#5, #9, #13): Further explore the role of EVs in the pathogenesis of AD, mainly involving key processes such as microglial activation, mitochondrial dysfunction, and pathological protein propagation; (2) Research on the therapeutic transformation of EVs (#0, #4, #8, #10, #11, #12): Focus on the preliminary application and exploration of EVs as drug delivery carriers and mesenchymal stem cell-derived EVs in the treatment of AD; (3) Methodologies and technical platforms (#2, #3, #14): Pay attention to basic methods for the isolation, purification, and identification of EVs, as well as technical strategies to improve the targeted uptake efficiency of EVs, providing methodological support for subsequent clinical translation; (4) Cross**—**research on neurodegenerative diseases (#1): Explore the comorbidity mechanisms between other neurodegenerative diseases such as Parkinson’s disease and Huntington’s disease and AD, as well as the common roles that EVs may play in them. In the keyword burst diagram from 2021 to 2026, the burst keywords mainly include multivesicular bodies, neurodegeneration, astrocyte—derived exosomes, small interfering RNA, clinical trials, and blood biomarkers, etc., further validating the robustness of the above clustering results (Figure 5D).

### 3.9 Validation using the PubMed database

To evaluate the robustness and comprehensiveness of the analysis results based on the WOSCC and Scopus databases, this study performed an equivalent retrieval strategy in the PubMed database for a consistency check, and a total of 1,614 articles were retrieved. Although there were slight differences in the literature coverage compared with WOSCC and Scopus, the publication time trend in the PubMed database was highly consistent with the former two (Figure 6A), and the top five countries in terms of the number of publications completely overlapped (Figure 6B). There were slight differences in the rankings of the top five institutions and authors in terms of the number of publications (Supplementary Tables 4, 5), which might be related to the specific indexing policies and journal coverage of each database. The high**—**frequency keywords and research hotspots showed a high degree of overlap (Figures 6C,D), such as Alzheimer’s disease; extracellular vesicle; mesenchymal stem cell; neuroinflammation; oxidative stress, biomarker; blood**—**brain barrier; diagnosis; neurodegeneration, etc. The above results jointly confirm that the core findings of this study show good robustness and reproducibility in multiple mainstream literature databases.

**Figure 6:**
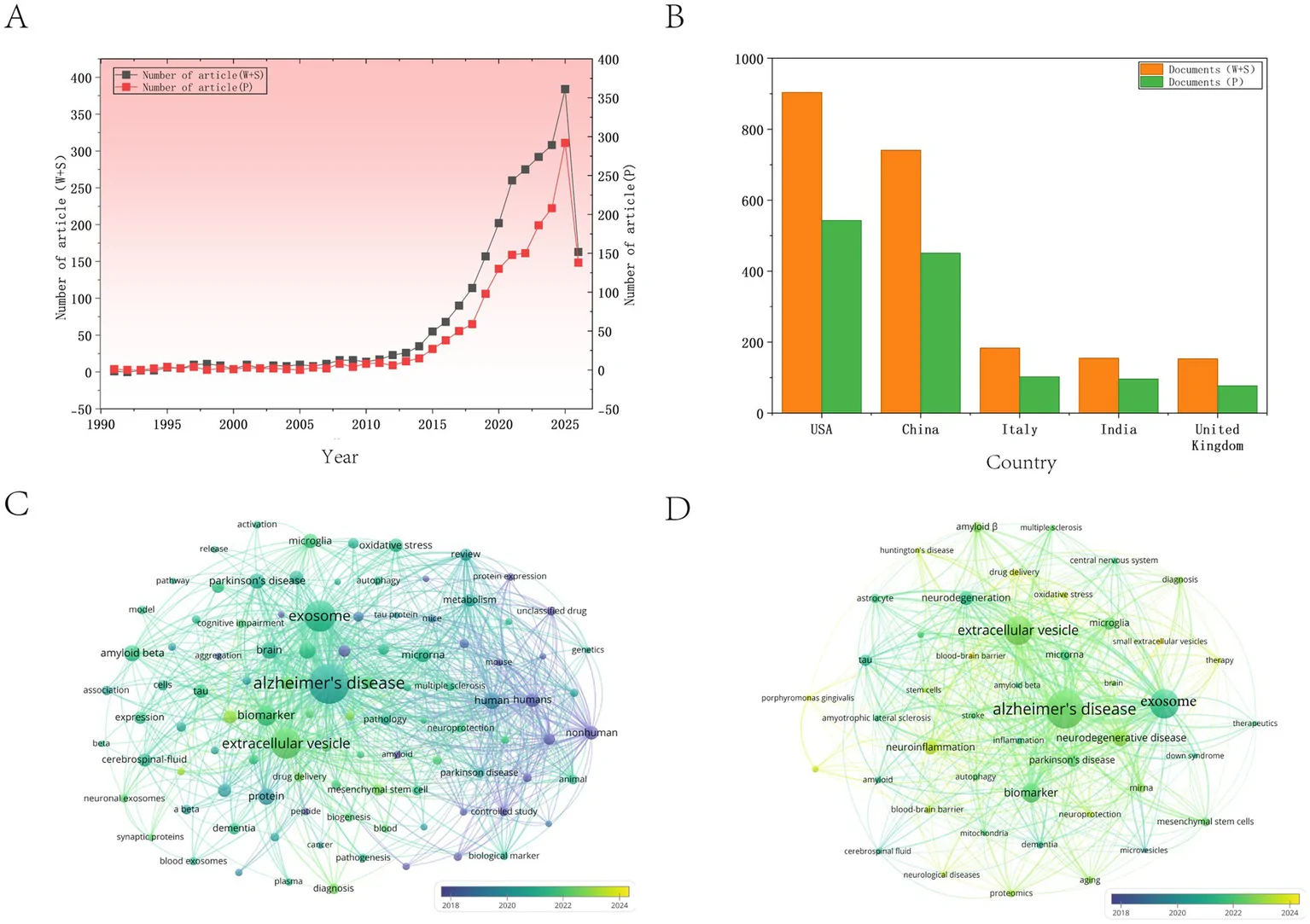
**(A)** Trends and annual measurements. **(B)** Country. **(C)** Keyword (WOS and Scopus). **(D)** Keyword (Pubmed).

## 4 Discussion

### 4.1 Summary of the research

This study employed bibliometric methods to systematically analyze the global research trends, topic evolution, and cooperation networks of EVs in the field of AD over the past 35 years. The results showed that since 2012, the activity of related research has been continuously increasing. Especially after 2020, the number of published papers has witnessed an explosive growth. This may be related to the COVID-19 pandemic (Fanelli et al., 2026), advances in high-throughput sequencing (Zhang X. et al., 2025) and single-vesicle analysis, and the increasing burden of Alzheimer’s disease (Hao and Chen, 2025). The United States leads in research productivity and academic influence. Although China has significantly improved its research productivity, the average influence level still needs to be enhanced. Judging from the recent high publication volume in journals such as International Journal of Molecular Sciences, Molecular Neurobiology, and Cells, molecular biology remains a major hotspot in this research field. Multiple institutions in the United States, including the National Institute on Aging, the University of California, San Francisco, and Boston University, have a high number of publications and high total link strength, indicating that they not only produce abundant scientific research results but also have extensive and far**—**reaching influence in knowledge dissemination and academic collaboration. The PubMed database further confirmed the robustness and reliability of the above research results.

### 4.2 Multifaceted mechanisms of EVs in AD pathology

The typical pathological features of AD include Aβ plaques, tau protein neurofibrillary tangles, and progressive neuronal loss (Li et al., 2026). In recent years, other pathogenic mechanisms have attracted increasing attention (Han et al., 2025). As repeatedly highlighted in Figures 4–6, emerging pathological mechanisms mediated by EVs—including neuroinflammation, oxidative stress, metabolic disorders, and mitochondrial dysfunction, etc. The appearance of these terms confirms that AD research is undergoing a paradigm shift from a single pathological perspective to a multi-mechanism parallel framework. As the core of cellular energy metabolism, under normal physiological conditions, EVs participate in the regulation of mitochondrial homeostasis by delivering functional mitochondria to recipient cells and mediating the lysosomal degradation of damaged mitochondria (Xing et al., 2026) However, in the pathological environment of AD, EVs-mediated mitochondrial dysfunction (such as oxidative stress, dysregulation of dynamics, and impaired mitophagy) plays a key role in disease progression (Zhao et al., 2026). Oxidative stress induced by mitochondrial dysfunction can exacerbate lipid peroxidation, damage the membrane structure, and promote Aβ production; while Aβ can further damage mitochondrial function, thus forming a vicious cycle that drives disease progression (Bajinka et al., 2026).

Central nervous system inflammation can also drive the occurrence and development of AD (Pascual et al., 2021). In the pathological microenvironment of AD, abnormally activated glial cells (such as microglia and astrocytes) can release a large number of EVs carrying pro—inflammatory microRNAs (miRNAs), pro**—**inflammatory factors (such as TNF-α) and other inflammatory mediators (Arbaciauskaite et al., 2026; Deng et al., 2025). As an important medium for intercellular communication, EVs spread inflammatory signals in the brain, promote the production and spread of pathological proteins, lead to the cascade amplification of neuroinflammation, and then promote the progression of AD (Twarowski and Herbet, 2023; Yu et al., 2025). Notably, extracellular vesicles secreted by human-induced pluripotent stem cell-derived neural stem cells can inhibit the pro-inflammatory cascade in disease-associated microglia in Alzheimer’s disease, thereby exerting neuroprotective effects (Madhu et al., 2024). A mouse experiment found that EVs in the feces of mice after stroke can carry pro—inflammatory signals. By damaging the integrity of the intestinal barrier and increasing intestinal permeability, they affect the central nervous system via the gut**—**brain axis, thereby exacerbating central nervous system inflammation and AD—related pathological changes (Kerr et al., 2025).

Metabolic abnormalities are also considered one of the key driving factors in the occurrence and development of AD (Li X. et al., 2025). Studies have shown that neuronal lipid metabolism disorders can induce autophagy and lysosomal dysfunction, which in turn promotes lysosomal stress and the release of mitochondria**—**derived EVs. After these EVs enter microglia, they activate the inflammatory pathway and drive neurodegeneration (Li X. et al., 2025). Another study found that the treatment with palmitic acid combined with high**—**glucose can induce insulin resistance in primary rat embryonic cortical neurons and human cerebral cortical stem cells, and affect the EVs secretion of glial cells. Thus, insulin resistance is more widely and indirectly amplified in the neural network, promoting the production and spread of pathological proteins and ultimately accelerating the progression of AD (Kim et al., 2023).

### 4.3 EVs-mediated comorbidity mechanism

Cluster and keyword analyses in Figures 4–6 identified the following core terms: neurodegeneration, Parkinson’s disease, multiple sclerosis, amyotrophic lateral sclerosis, cardiovascular disease, *Porphyromonas gingivalis*, among others. The comorbidity between AD and other neurodegenerative diseases, as well as systemic diseases, is receiving increasing attention. In other neurodegenerative diseases such as PD, ALS, and MS, despite distinct pathological features, common molecular mechanisms exist in protein misfolding, pathological protein propagation, mitochondrial dysfunction, neuroinflammation, and oxidative stress. The elucidation of these shared mechanisms provides a theoretical foundation for the cross-fertilization of diagnostic techniques and therapeutic strategies across neurodegenerative diseases (Pink et al., 2021; Wiersema et al., 2024).

The comorbidity between AD and systemic diseases, including cardiovascular disease, chronic kidney failure, liver cirrhosis, and bacterial infection, is likewise gaining increasing attention. These systemic diseases are not only associated with an elevated risk of AD but may also accelerate disease progression through overlapping pathological mechanisms. Epidemiological evidence indicates that individuals with multiple cardiometabolic diseases have a 49% higher risk of developing AD (Zhang J. et al., 2025). Rodent models of myocardial injury have demonstrated that cardiac injury induces significant neuroinflammation in the brain, with the underlying mechanism involving the secretion of miRNA-21-loaded extracellular vesicles by the injured myocardium, which directly activate brain microglia and subsequently trigger neuroinflammatory responses (Li Q. et al., 2025). Conversely, AD-related pathological proteins (Aβ, tau) can also be loaded into EVs, cross the damaged blood–brain barrier into the peripheral circulation, and accumulate in the myocardium, thereby inducing myocardial dysfunction and vascular endothelial injury (Al-Kuraishy et al., 2025).

An increasing body of evidence suggests that bacterial outer membrane vesicles (OMVs), a class of EVs secreted by Gram-negative bacteria, originate from the oral cavity, olfactory, and upper respiratory tract microecological niches and can carry bacterial toxins, nucleic acids, and proteins (Han et al., 2019). Unlike eukaryotic EVs, OMVs are derived from the bacterial outer membrane and are typically enriched in lipopolysaccharides (LPS), outer membrane proteins, and periplasmic contents. These OMVs enter the central nervous system (CNS) via the bloodstream and direct neural pathways, including the olfactory and trigeminal nerves, thereby participating in the pathological progression of AD (Delbaz and St John, 2026). *Porphyromonas gingivalis*, a key periodontal pathogen, produces OMVs enriched with pro-inflammatory factors such as gingipains (GPs) and LPS (Liu et al., 2024). These EVs may enter the CNS through either the trigeminal nerve or the periodontal blood circulation, subsequently inducing an inflammatory response in the hippocampus, downregulating the expression of key proteins such as brain-derived neurotrophic factor (BDNF), and ultimately leading to cognitive decline (Ma et al., 2023).

### 4.4 Precise diagnosis of Alzheimer’s disease using EVs

In recent years, the diagnostic value of EVs in AD has also become one of the research hotspots. Both the cluster analysis and keyword analysis in Figures 4–6 identify the following core words: accurate diagnosis, brain**—**derived extracellular vesicle, peripheral blood biomarker, diagnostic alchemy. Since the onset of AD is insidious, screening out highly sensitive and specific biomarkers before the appearance of obvious clinical symptoms is of great significance for the early diagnosis of AD and the prediction of disease progression (Xing et al., 2021). In the early stage of AD, significant changes occur in brain—derived EVs (nEVs) and their carried biomarker profiles (including pathological proteins, miRNAs, metabolites, etc.), which can reflect the degree of nerve damage and the stage of pathological development (Arif et al., 2025; Sun et al., 2023; Vogrinc et al., 2021). In addition, because EVs can penetrate the blood**—**brain barrier, researchers have found that obtaining EVs and their contents from the central nervous system in the peripheral circulation can also serve as a new strategy for the early diagnosis of AD (Fiandaca et al., 2015; Singh et al., 2026). It is worth noting that multiple studies have shown that peripheral blood**—**derived EVs (pEVs) have a similar diagnostic efficacy for AD and mild cognitive impairment compared with nEVs (de la Monte et al., 2025; Winston et al., 2016).

In addition to plasma-derived exosomes, saliva, urine, and tears have gained attention due to their non-invasive nature and ease of collection. Aβ oligomers/fibrils and phosphorylated tau are significantly elevated in salivary exosomes of patients with cognitive impairment and AD (Rani et al., 2021). Ryu et al. (2023) found that miR-485-3p in human saliva-derived EVs closely correlates with cerebral Aβ deposition and shows high diagnostic accuracy for Aβ-PET positivity. A 2026 review highlighted the potential of salivary biomarkers, including Aβ, tau, lactoferrin, and exosomes, for AD diagnosis (Świątek et al., 2026). Regarding urine, Song et al. (2020) first reported that Annexin 2 and Clusterin in urinary exosomes of 5XFAD AD mice decrease with AD progression. Zhou et al. (2022) found that lipid metabolism- and Aβ clearance-related proteins are enriched in early-stage urinary exosomes from J20 AD mice. Additionally, an ISEV Urine Task Force position paper provided a standardized framework for the isolation, characterization, and clinical translation of urinary EVs (Erdbrügger et al., 2021). In tears, a review of five studies suggested that changes in protein, lipid, or metabolite concentrations may aid AD detection (Fotovat-Ahmadi et al., 2025). Schmeetz et al. (2025) first examined AD-related biomarkers in tear-derived EVs and found that EV-bound pTau-181 was significantly higher than the free form (1.7-fold, *p* = 0.016), suggesting that tear-derived EVs may serve as potential tools for non-invasive AD diagnosis. However, further studies are needed to validate the clinical translation of these detection methods.

In terms of AD disease staging, EVs-derived biomarkers show the potential to distinguish different stages of the disease course. N-(1-Carboxymethyl)-L-Lysine (CML) in pEVs of AD patients can distinguish early-stage from moderate-stage AD. The level of CML is higher in the early stage of AD, while it is lower in the mid-stage of AD (Haddad et al., 2019). Another study found that in pEVs of MCI patients, the level of amyloid precursor protein (APP) and the ratio of pTau181/tTau increase earlier, while the levels of Aβ42 and pTau-T181 in pEVs of AD patients increase relatively late (Perrotte et al., 2020). The above findings suggest that the combined detection of EV marker profiles at different stages is expected to achieve dynamic monitoring and accurate staging of the AD disease progression.

In terms of disease discrimination, the biomarkers carried by EVs also show the potential to distinguish different neurodegenerative diseases (Chai et al., 2024). In pEVs, the expression levels of hsa-miR-451a and hsa-miR-21-5p in samples from patients with AD are significantly lower than those in patients with dementia with Lewy bodies (DLB), suggesting that these two miRNAs may serve as potential biomarkers for the auxiliary differential diagnosis of AD and DLB (Gámez-Valero et al., 2019). In addition, long non-coding RNAs (RP11-462G22.1, PCA3) and messenger RNA transcripts (including APP, α-synuclein, Tau, neurofilament light chain) are differentially expressed in nEVs from patients with Parkinson’s disease (PD) and AD, and they can also serve as potential biomarkers for the auxiliary differential diagnosis of these two types of neurodegenerative diseases (Gui et al., 2015).

### 4.5 EV cargo and tetraspanins

With their ability to cross biological barriers, low immunogenicity, and excellent biocompatibility, EVs show promising therapeutic potential for AD (Mehdizadeh et al., 2025). During their formation, EVs can naturally carry or be artificially loaded with nucleic acids (miRNA, mRNA, siRNA), proteins, small-molecule drugs, and lipid signaling molecules. These cargoes exhibit enhanced therapeutic effects when delivered by EVs compared with free cargoes, making this an emerging area of research in AD treatment (Figures 4–6) (Zhang Y. et al., 2025). Alvarez-Erviti et al. (2011) successfully delivered siRNA to the mouse brain using targeted EVs, pioneering the use of EV-loaded RNAs for targeted therapy in central nervous system diseases. Kim et al. (2025) discovered that cellular proteasomes could be efficiently packaged into EVs and secreted as intact 20S particles with preserved enzymatic activity, which promotes the degradation of overexpressed Tau protein without disrupting overall cellular proteolytic balance. When curcumin is loaded into engineered EVs, it is efficiently delivered to neuronal mitochondria, significantly reducing Aβ-induced mitochondrial membrane potential decline, excessive reactive oxygen species production, and mitochondrial dysfunction, thereby preserving neuronal function (Ding et al., 2025). Lipid cargoes from adipose-derived EVs, particularly lysophosphatidylcholine and sphingomyelin species, can alter the aggregation kinetics of Aβ40 and Aβ42 *in vitro* in a lipid type- and concentration-dependent manner (Yang et al., 2025).

Tetraspanins, a protein family with four transmembrane domains, comprise 33 members in mammal (Garcia-España et al., 2008). In the context of identifying and characterizing EVs, tetraspanins such as CD81, CD9, and CD63 are core markers recommended by the MISEV2023 guidelines (Journal of Extracellular Vesicles, 2024). Beyond serving as markers, tetraspanins are increasingly recognized for their regulatory roles in AD pathology (Rubinstein et al., 2025). Tspan12, for instance, facilitates the maturation of ADAM10 (a disintegrin and metalloprotease 10) and enhances its α-cleavage of APP (Xu et al., 2009). Members of the TspanC8 subfamily, including Tspan5 and Tspan15, differentially influence the endocytosis and surface retention of ADAM10 (Eschenbrenner et al., 2020). In AD patients, Tspan6 is upregulated in the prefrontal cortex, causing an accumulation of APP C-terminal fragments (APP-CTF) by increasing the secretion of EVs containing APP-CTF and disrupting autophagosome-lysosome fusion (Guix et al., 2017). This evidence suggests that tetraspanins have evolved from being mere EV markers to potential regulators of AD pathology, although their specific mechanisms in AD require further investigation.

### 4.6 Clinical research advances

Currently, research on EVs in the diagnosis and treatment of AD remains primarily in the pre**—**clinical stage, mostly focusing on mouse models and tissue levels, with relatively limited clinical translation studies. Most clinical trials are still in the registration or ongoing phase, and only a few have published their research results. In treatment**—**related clinical trials, the main administration methods of EVs include nasal spray, intracerebral injection, and intravenous injection. Among them, nasal administration has demonstrated unique advantages due to its non**—**invasiveness, ability to bypass the blood**—**brain barrier, and effective distribution in the brain parenchyma, and has become the preferred administration route for multiple clinical trials (Rossi et al., 2025; Shen et al., 2026). Ruijin Hospital Affiliated to Shanghai Jiao Tong University School of Medicine has conducted a Phase I/II trial (NCT04388982) for patients with mild to moderate AD. This trial adopted a “3 + 3” dose escalation design. The subjects were successively assigned to three dose groups and received intranasal administration of EVs derived from allogeneic adipose-derived mesenchymal stromal cells. Studies have confirmed that this therapy has good safety and tolerability, can improve patients’ cognitive function, and its neuroprotective effect can delay the atrophy of the hippocampus (Xie et al., 2023).

A rigorously designed randomized, double-blind, placebo-controlled study (NCT07457125) initiated by Beijing Xuanwu Hospital in March 2026 adopted a two-phase design, including a dose escalation phase and an expanded cohort phase, aiming to evaluate the efficacy and safety of intranasal administration of EVs derived from umbilical cord mesenchymal stromal cells in the treatment of patients with mild to moderate AD. The trial is expected to be completed in 2028, and its results will provide an important reference for the clinical transformation of EVs derived from umbilical cord mesenchymal stromal cells (Trabulo et al., 2026).

Prodromos and colleagues conducted a series of clinical studies and reported the treatment results of MSC—EVs in various neurodegenerative diseases. This study included 18 patients with amyotrophic lateral sclerosis, Kennedy’s disease, congenital myasthenic syndrome, and Lewy body dementia. After nasal administration, all patients except one showed significant improvement in muscle strength, and no adverse events were reported (Prodromos et al., 2025). Another clinical study on 86 patients with ALS, LBD, and PD evaluated the safety and preliminary efficacy of MSC—EVs/secretome via the intranasal administration route. The results showed that up to 77% (67/86) of the patients had a positive clinical response to at least one product, and no serious adverse events were reported (Prodromos et al., 2026).

In clinical trials related to diagnosis, Yingzhi Zhang’s team published a study with existing results. The team established a rapid and non-invasive single vesicle analysis method based on EV-CATCH technology combined with nano-flow cytometry. The results show that this method demonstrates extremely high accuracy in differentiating the status of Aβ-PET and diagnosing MCI and AD. This technology only requires a trace amount of serum, and the cost of single-sample consumables is low. It also has the advantages of being non-invasive, economical and efficient, providing a practical and feasible technical solution for large-scale early screening of AD (Zhang Y. et al., 2026).

Another registered MINERVA trial (NCT06869135) analyzes the molecular fingerprints of saliva and saliva-derived extracellular vesicles through Raman spectroscopy and quantitatively analyzes disease-related proteins in combination with ultra-sensitive detection technology, aiming to achieve early identification and phenotypic typing of Alzheimer’s disease, Parkinson’s disease, atypical Parkinson’s syndrome, pre-Parkinson’s disease, and mild cognitive impairment. This trial adopts a design that combines cross-sectional and 12-month longitudinal follow-up. It will be launched in January 2025 and is expected to be completed in December 2027. This study will provide a new strategy for non-invasive and low-cost early screening of neurodegenerative diseases.

In addition, a cross-sectional and exploratory diagnostic study (ChiCTR2400079450) initiated by the Xi’an Regional Medical Laboratory Center aims to isolate brain-derived extracellular vesicles from serum, identify differentially expressed protein or RNA markers, and evaluate their clinical diagnostic capabilities for Alzheimer’s disease, mild cognitive impairment, and non-AD dementia. The enrollment of this study was completed from December 2023 to November 2024, and it is currently in the data analysis stage, with the research results yet to be published.

### 4.7 Prospects and challenges

As a natural nanoscale intercellular communication carrier, EVs have demonstrated unique advantages in the treatment of AD: (1) The ability to cross the blood–brain barrier: EVs can act as a carrier to penetrate the blood–brain barrier and deliver drugs to the CNS (Alvarez-Erviti et al., 2011); (2) Multi-target synergistic intervention: It can carry multi-functional molecules such as siRNA, curcumin, resveratrol, and neurotrophic molecules, and conduct multi-target intervention targeting the complex pathological mechanisms of AD (Du et al., 2025; Wei et al., 2024); (3) Low immunogenicity and high biocompatibility: Alleviate immune rejection reactions; (4) Potential for engineering transformation: Through surface modification or load loading, targeted delivery and on-demand release can be achieved to enhance the level of precision treatment (Huang et al., 2024).

Despite its promising prospects, the application of EVs in the treatment of AD still faces many challenges: (1) Large-scale production and standardization: There is a lack of unified quality control standards for the separation and purification of EVs (Welsh et al., 2024; Witwer et al., 2017); (2) Heterogeneity and batch differences: Different cell sources and different culture conditions lead to significant differences in the composition and function of EVs (Willms et al., 2018); (3) Targeted efficiency needs to be improved: The enrichment efficiency of EVs in the brain still requires further optimization (Huang et al., 2024); (4) Clinical transformation bottleneck: The application of EVs in the diagnosis and treatment of AD lacks large-scale research and new safety evaluations (Reiner et al., 2017); (5) Biological sample selection: The potential value of extracellular vesicles from saliva, urine, and tears in AD still requires further studies and clinical trials for validation. Future studies may also further explore EVs from other biological fluids (Cao et al., 2025).

## 5 Conclusion

This study conducted a comprehensive bibliometric and visual analysis of the relevant research on EVs in the AD field from 1991 to 2026. Through a systematic review of 2,626 documents, the results show that the number of publications in this field has generally been on the rise year by year, especially experiencing explosive growth after 2020. The United States and China are at the forefront of the world in terms of the number of published papers and academic influence, while showcasing influential journals, core institutions and highly contributing authors in this field. The co-occurrence and co-citation analysis of key words indicates that the research of EVs in AD has shifted from a single pathological mechanism to a multi-mechanism parallel framework. Emerging pathological mechanism, oxidative stress, peripheral blood cell, stem cell, mesenchymal stromal cells, targeted Topics such as drug delivery, miRNA, and clinical trails have gradually become emerging research frontiers. The PubMed database further confirmed the robustness and reliability of the above research results. In conclusion, this study, through rigorous methods and multi-framework analysis, comprehensively reveals the research trends and hotspots of EVs in the field of AD, providing important reference basis and direction guidance for the basic research and clinical transformation of EVs in the field of AD.

## Data Availability

The datasets presented in this study can be found in online repositories. The names of the repository/repositories and accession number(s) can be found in the article/Supplementary material.
