# Supplementary material for: Extracellular vesicles and Alzheimer’s disease: hotspots, trends, and future directions—a tri-database bibliometric analysis
Source: Front Aging Neurosci. 2026 Oct 26;18:1951959. doi: 10.3389/fnagi.2026.1951959 (PMC13745720; doi:10.3389/fnagi.2026.1951959)
Supplement: Supplementary file 1 [file Data_Sheet_1.DOC]

Table 1

| Database | Search strategy |
| --- | --- |
| WOS | TS=("Alzheimer*" OR "Alzheimer disease" OR "Alzheimer's disease" OR "Senile Dementia") AND TS=("extracellular vesicle*" OR "vesicle, extracellular" OR "vesicles, extracellular" OR "endovesicles" OR "exovesicles" OR "exosomes" OR "apoptotic bodies" OR "apoptotic body" OR "bodies, apoptotic" OR "body, apoptotic" OR "Cell-Derived Microparticles" OR "microvesicil*" OR "exocytosis vesicles" OR "secretion vesicles" OR "lipid vesicles" OR "membrane vesicles") |
| Scopus | ( TITLE-ABS-KEY ( "Alzheimer*" OR "Alzheimer disease" OR "Alzheimer's disease" OR "Senile Dementia" ) AND TITLE-ABS-KEY ( "extracellular vesicle*" OR "vesicle, extracellular" OR "vesicles, extracellular" OR "exovesicles" OR "exovesicle" OR "exosomes" OR "apoptotic bodies" OR "apoptotic body" OR "bodies, apoptotic" OR "body, apoptotic" OR "Cell-Derived Microparticles" OR "microvesicil*" OR "exocytosis vesicles" OR "secretion vesicles" OR "lipid vesicles" OR "membrane vesicles" ) ) AND PUBYEAR < 2027 AND NOT PUBDATETXT ( "May 2026" OR "June 2026" OR "July 2026" OR "August 2026" OR "September 2026" OR "October 2026" OR "November 2026" OR "December 2026" ) |
| Pubmed | ("Alzheimer*"[Title/Abstract] OR "Alzheimer disease"[Title/Abstract] OR "Alzheimer's disease"[Title/Abstract] OR "Senile Dementia"[Title/Abstract]) AND ("extracellular vesicle*"[Title/Abstract] OR "vesicle, extracellular"[Title/Abstract] OR "vesicles, extracellular"[Title/Abstract] OR "endovesicles"[Title/Abstract] OR "exovesicles"[Title/Abstract] OR "exosomes"[Title/Abstract] OR "apoptotic bodies"[Title/Abstract] OR "apoptotic body"[Title/Abstract] OR "bodies, apoptotic"[Title/Abstract] OR "body, apoptotic"[Title/Abstract] OR "Cell-Derived Microparticles"[Title/Abstract] OR "microvesicil*"[Title/Abstract] OR "exocytosis vesicles"[Title/Abstract] OR "secretion vesicles"[Title/Abstract] OR "lipid vesicles"[Title/Abstract] OR "membrane vesicles"[Title/Abstract]) |

Table 2. The top 10 articles in terms of citation counts

| RANK | Article | Author | Citations | Year | Type |
| --- | --- | --- | --- | --- | --- |
| 1 | Delivery of siRNA to the mouse brain by systemic injection of targeted exosomes | Lydia Alvarez-Erviti | 4138 | 2011 | Article |
| 2 | Exosomes: Current knowledge of their composition, biological functions, and diagnostic and therapeutic potentials | [Alexander V. Vlassov](https://www.sciencedirect.com/author/6701605991/alexander-v-vlassov) | 1778 | 2012 | Review |
| 3 | Stabilization of α-Synuclein Secondary Structure upon Binding to Synthetic Membranes | [W. Sean Davidson](https://www.jbc.org/article/S0021-9258(18)46153-4/fulltext) | 1413 | 1998 | Article |
| 4 | Extracellular vesicle in vivo biodistribution is determined by cell source, route of administration and targeting | [Oscar P. B. Wiklander](https://isevjournals.onlinelibrary.wiley.com/authored-by/Wiklander/Oscar+P.+B.) | 1400 | 2015 | Article |
| 5 | Depletion of microglia and inhibition of exosome synthesis halt tau propagation | Hirohide Asai | 1304 | 2015 | Article |
| 6 | Alzheimer's disease β-amyloid peptides are released in association with exosomes | [Lawrence Rajendran](https://www.pnas.org/doi/full/10.1073/pnas.0603838103#con1) | 1107 | 2006 | Article |
| 7 | Axonopathy and transport deficits early in the pathogenesis of Alzheimer's disease | [Gorazd B Stokin](https://pubmed.ncbi.nlm.nih.gov/?sort=date&term=Stokin+GB&cauthor_id=15731448) | 1082 | 2005 | Article |
| 8 | Microglia in neurodegenerative diseases: mechanism and potential therapeutic targets | [Chao Gao](https://www.nature.com/articles/s41392-023-01588-0#auth-Chao-Gao-Aff1) | 1059 | 2023 | Review |
| 9 | Exosome-associated Tau Is Secreted in Tauopathy Models and Is Selectively Phosphorylated in Cerebrospinal Fluid in Early Alzheimer Disease | [Sudad Saman](https://www.jbc.org/article/S0021-9258(20)48193-1/fulltext) | 798 | 2012 | Article |
| 10 | Exosomes and microvesicles: extracellular vesicles for genetic information transfer and gene therapy | [Yi Lee](https://academic.oup.com/hmg/article-abstract/21/R1/R125/javascript:;) | 776 | 2012 | Article |

Table 3. The top 10 articles in terms of co-citation counts.

| RANK | Article | Author | Citations | Year | Total link strength | Source |
| --- | --- | --- | --- | --- | --- | --- |
| 1 | Identification of preclinical Alzheimer's disease by a profile of pathogenic proteins in neurally derived blood exosomes: A case-control study | Fiandaca MS | 435 | 2015 | 4467 | Alzheimer's & Dementia |
| 2 | Alzheimer's disease β-amyloid peptides are released in association with exosomes | Rajendran L | 378 | 2006 | 4030 | Proceedings of the National Academy of Sciences (PNAS) |
| 3 | Delivery of siRNA to the mouse brain by systemic injection of targeted exosomes | Alvarez-Erviti L | 288 | 2018 | 2911 | Nature Biotechnology |
| 4 | Depletion of microglia and inhibition of exosome synthesis halt tau propagation. | Asai H | 285 | 2015 | 3201 | Nature Neuroscience |
| 5 | Minimal information for studies of extracellular vesicles 2018 (MISEV2018) | Théry C | 281 | 2012 | 2248 | Journal of Extracellular Vesicles |
| 6 | Exosome-associated Tau is secreted in tauopathy models. | Saman S | 259 | 2018 | 3031 | Journal of Biological Chemistry |
| 7 | Exosome-mediated transfer of mRNAs and microRNAs | Valadi H | 223 | 2016 | 2016 | Nature Cell Biology |
| 8 | The biology, function, and biomedical applications of exosomes. | [Raghu Kalluri](https://pubmed.ncbi.nlm.nih.gov/?sort=date&term=Kalluri+R&cauthor_id=32029601) | 218 | 2014 | 1699 | Science |
| 9 | Extracellular vesicles: exosomes, microvesicles, and friends. | Raposo G | 215 | 2011 | 2052 | Journal of Cell Biology |
| 10 | Shedding light on the cell biology of extracellular vesicles. | van Niel G | 210 | 2012 | 1864 | Nature Reviews Molecular Cell Biology |

Table 4.The top 5 institutions in terms of publications from the PubMed dataset

| RANK | Institution（P） | Documents |
| --- | --- | --- |
| 1 | University Of California | 49 |
| 2 | National Institute On Aging | 40 |
| 3 | Boston University School Of Medicine | 23 |
| 4 | Harvard Medical School | 22 |
| 5 | Capital Medical University | 20 |

Table 5.The top 5 authors in terms of publications from the PubMed dataset

| RANK | Author（Pubmed） | Documents |
| --- | --- | --- |
| 1 | Kapogiannis, Dimitrios | 57 |
| 2 | Goetzl, Edward J | 28 |
| 3 | Ikezu, Tsuneya | 26 |
| 4 | Mustapic, Maja | 26 |
| 5 | Hill, Andrew F | 18 |
